# Prevalence and Associated Risk Factors of Self-Harm Among Health Care Workers: Protocol for Systematic Review and Meta-Analysis

**DOI:** 10.2196/67059

**Published:** 2025-09-17

**Authors:** Nor Asiah Muhamad, Nur Hasnah Maamor, 'Izzah 'Athirah Rosli, Tengku Puteri Nadiah Tengku Baharudin Shah, Nurul Hidayah Jamalluddin, Fatin Norhasny Leman, Nik Athirah Farhana Nik Azhan, Shiao Ling Ling, Norliza Chemi, Suria Hussin, Fariza Yahya, Norli Abdul Jabbar, Nurashikin Ibrahim

**Affiliations:** 1 Sector for Evidence-based Healthcare National Institutes of Health Ministry of Health Selangor Malaysia; 2 Department of Psychiatric and Mental Health Kajang Hospital Ministry of Health Selangor Malaysia; 3 Department of Psychiatry and Mental Health Raja Perempuan Zainab II Hospital Ministry of Health Kelantan Malaysia; 4 National Centre of Excellence in Mental Health Ministry of Health Cyberjaya Malaysia

**Keywords:** self-harm, health care workers, protocol, systematic review, meta-analysis

## Abstract

**Background:**

Self-harm is a major public health concern, with prevalence increasing worldwide, particularly after the COVID-19 pandemic and associated lockdown restrictions. Health care workers (HCWs) face various challenges, such as pressures of social and familial responsibilities, a lack of integration within the profession, heavier workload, bullying at the workplace, and limited support in the workplace, that impact their mental health and often lead to self-harm.

**Objective:**

We aim to synthesize the evidence on the pooled prevalence of self-harm worldwide and identify risk factors for self-harm among HCWs.

**Methods:**

We will conduct a systematic review of observational and experimental studies that investigated the overall prevalence of self-harm among HCWs. We will search the PubMed, PsycINFO, Embase, and CINAHL databases for eligible articles from inception until March 2025 using specific search terms developed using the population, exposure, comparison, and outcome framework. Study selection and reporting will follow the PRISMA (Preferred Reporting Items for Systematic Reviews and Meta-Analyses) and the Meta-Analysis of Observational Studies in Epidemiology guidelines. We will contact the corresponding author via email if the required data are not available in the article. After completing the article search, duplicate records will be removed. Titles and abstracts will then be screened according to the inclusion and exclusion criteria, followed by retrieval of the full texts for detailed screening. All the required data for the review, such as names of authors, publication year, prevalence of self-harm, type of profession, associated risk factors to self-harm, and others, will be extracted using a standardized data extraction form. The quality of the studies will be assessed using the Joanna Briggs Institute guidelines based on the study design. Random-effects meta-analysis will be used to derive the pooled prevalence using Stata (version 17.0) software. We will conduct a subgroup meta-analysis on sex, regions, and the type of profession (physicians or nurses). We will also examine the association of risk factors of self-harm with sociodemographic factors to observe their relationship. Both analyses will be performed using RevMan software. Publication bias will be examined using the funnel plot and Egger test.

**Results:**

Data analysis is expected to be completed by August 2025, and manuscript preparation is expected to be completed by October 2025. This review is expected to be completed and published by January 2026.

**Conclusions:**

We will provide a comprehensive synthesis of the overall prevalence of self-harm among HCWs. We will also provide important information to develop effective strategies for preventing and managing self-harm among HCWs.

**Trial Registration:**

PROSPERO CRD42024581791; https://www.crd.york.ac.uk/PROSPERO/view/CRD42024581791

**International Registered Report Identifier (IRRID):**

PRR1-10.2196/67059

## Introduction

### Background

Self-harm refers to the deliberate initiation of behaviors, for example, self-cutting, with the intention of causing harm to oneself [1]. It is also reported as an early manifestation of suicide [2] or a short-term risk factor for future attempted and completed suicide [3]. Self-harm tends to be underreported, although this issue affects countries and societies worldwide [4]. It had been reported that in 2015, approximately 575,000 people attended a hospital for injuries due to self-harm in the United States [5]. During the COVID-19 pandemic in 2021, an increase in psychiatric illness cases [6] was observed, where the cases were different based on the working categories. This was due to the loss of jobs and struggle for income among the low socioeconomic group [7]. This led to an increase in depression; anxiety; insomnia; substance abuse; and fear of contagion, losing loved ones, and transmitting infection to loved ones. These factors have contributed to an increased chance of self-harm [8].

Medical and health professionals or health care workers (HCWs) are often required to work for an extended time, which may contribute to long-term stressful work conditions, affecting both physical and mental health. In addition, moral injury was reported to be significantly associated with the risk of self-harm among HCWs, manifesting as symptoms of self-guilt, self-denial, self-blame, sadness, and anger [9]. Besides that, an unprecedented workload, tension in physician-patient relationships, and moral distress led to a higher risk of burnout and moral injury to HCWs. Therefore, addressing the high level of psychological impact and self-harm ideation in this population is crucial and has become an important public health concern that affects all sectors of society [9]. Before the pandemic, HCWs were reported to have higher rates of depression, anxiety, traumatic stress conditions, suicidal ideation, and self-harm compared to the general population, and the prevalence increased during the COVID-19 pandemic [10]. For example, the prevalence of self-harm among HCWs during the COVID-19 pandemic was 46.9% in Australia [7], 38% in China [9], and 13% in the United Kingdom [11]. The prevalence of self-harm among HCWs was reported to be 5.3% before the pandemic compared to 9.3% after the pandemic [12,13].

This shows that the issue of HCWs and self-harm has become an important public health concern that affects all sectors of society [14]. Although a few reviews have reported on self-harm among HCWs, no single systematic review has reported on this issue during, before, and after the COVID-19 pandemic. This comparison is needed as it was previously stated in a few articles [7,9,11] that the increase in prevalence of self-harm among HCWs after the impact of the COVID-19 pandemic is a growing concern for suicide risk among HCWs.

### This Study

Therefore, we will conduct a systematic review and meta-analysis to synthesize the evidence on the global overall or pooled prevalence of self-harm and risk factors associated with HCWs. We will also perform subgroup analyses based on professional groups, geographic regions (Asia, Africa, Europe, Oceania, and North and South America), and settings (urban vs rural). Furthermore, we seek to identify the associated risk factors of self-harm among HCWs. The findings will be used to identify further research and prevention efforts.

## Methods

### Study Design

This protocol is registered with PROSPERO (CRD42024581791). The review will be conducted and reported following the PRISMA (Preferred Reporting Items for Systematic Reviews and Meta-Analysis) protocol [15] and Observational Studies in Epidemiology guideline.

We will include all empirical study designs, including observational, for example, case-control, cohort, and cross-sectional, to capture all desired outcomes. No restrictions will be imposed on the date of publication and sample size.

### Study Area

We will include all published studies with information on self-harm among HCWs globally and later divide the studies according to the regions and ages. The period before the COVID-19 pandemic is defined as the period before March 2020. We will examine the methodology section to determine when the data collection occurred. Conversely, the period after the COVID-19 pandemic refers to the period following March 2020.

### Type of Participants

We will include primary studies that investigated self-harm or nonsuicidal injury (or other synonyms) among HCWs aged 18 years and above. In addition, if the required data are not available in the article, we will contact the corresponding author to obtain clarification and data from that article.

We will exclude studies that do not involve HCWs and include participants aged less than 18 years. If a study includes medical students, their data will also be excluded from this study due to different work responsibilities. If we find multiple articles with the same data, then we will use the study with the largest sample size. The findings will be presented in tables and figures.

### Type of Outcome Measures

The primary outcome will be the pooled prevalence of self-harm among HCWs before and after the COVID-19 pandemic. The secondary outcomes will be the risk factors associated with self-harm based on the 3 domains: psychological factors, work-related factors, and personal factors.

### Searching Methods for the Identification of Studies

We will search the articles in 4 different databases: PubMed, PsycINFO, Embase, and CINAHL for any eligible articles from inception until July 2025. There is no restriction on the publication date; however, the language will be restricted to English. We will include primary studies and exclude conference proceedings, abstracts, editorials, and comments. We will include studies discussing self-harm among HCWs only.

### Search Strategy

The population, exposure, comparison, and outcome framework will be used during the article search. “Population” refers to HCWs (physicians and nurses), “exposure” refers to self-harm or nonsuicidal self-injury, and “outcome” refers to the prevalence and risk factors associated with self-harm among HCWs. The “comparisons” aspect is not included in this review. “OR” will be used to widen the keyword search and “AND” to narrow the search. The details of the proposed search terms for all the databases are listed in Table 1 and Multimedia Appendix 1.

**Table 1:** Detailed proposed search terms for all databases.

Number	Search terms
#1	Health care worker*
#2	Health care staff*
#3	Health care professional*
#4	Health care provider*
#5	Health care personnel*
#6	HCW
#7	Nurse*
#8	Doctor*
#9	Physician*
#10	Surgeon*
#11	Therapist*
#12	Houseman
#13	House officer
#14	Medical officer
#15	nurs*
#16	midwi*
#17	registered nurse
#18	RN
#19	licensed practical nurse
#20	LPN
#21	Medical assistant*
#22	Paramedic*
#23	Pharmacist*
#24	hospital staff*
#25	General practitioner*
#26	GP
#27	Frontlin*
#28	Employer*
#29	Employee*
#30	Medical personnel*
#31	Medical professional*
#32	#1 OR #2 OR #3 OR #4 OR #5 OR #6 OR #7 OR #8 OR #9 OR #10 OR #11 OR #12 OR #13 OR #14 OR #15 OR #16 OR #17 OR #18 OR #19 OR #20 OR #21 OR #22 OR #23 OR #24 OR #25 OR #26 OR #27 OR #28 OR #29 OR #30 OR #31
#33	self overdos*
#34	Deliberate self harm
#35	DSH
#36	Self inflict*
#37	Self harm*
#38	Self mutilat*
#39	Self poison*
#40	Self injur*
#41	automutilat*
#42	ligature strangulate*
#43	self hurt*
#44	self lacerat*
#45	self strang*
#46	self hang*
#47	self neglect*
#48	self destruct*
#49	self immolat*
#50	self violen*
#51	Self-Injurious Behav*
#52	#33 OR #34 OR #35 OR #36 OR #37 OR #38 OR #39 OR #40 OR #41 OR #42 OR #43 OR #44 OR #45 OR #46 OR #47 OR #48 OR #49 OR #50 OR #51
#53	Prevalen*
#54	incidence
#55	Number of cases
#56	Risk factor*
#57	Associated factor*
#58	Predisposing factor*
#59	Factor*
#60	Predictor*
#61	Financial problem
#62	Marital status
#63	Family crisis
#64	Family problem
#65	Mental health problem
#66	Work related problem
#67	Physical health problem
#68	Substance use problem
#69	Alcohol problem
#70	Drug problem
#71	Poor support system
#72	#53 OR #54 OR #55 OR #56 OR #57 OR #58 OR #59 OR #60 OR #61 OR #62 OR #63 OR #64 OR #65 OR #66 OR #67 OR #68 OR #69 OR #70 OR #71
#73	#32 AND #52 AND #72

### Data Collection and Analysis

#### Selection of Studies

Three review authors (NAM, TPNTBS, and NHJ) will independently screen for titles and abstracts to identify the potential studies to be included. The studies will be identified and coded as “retrieve” (studies to be included, with potential to be included, or unclear) or “do not retrieve” (studies to be excluded). We will also identify and exclude duplicate studies. If there is an overlap in the included studies, the authors will read the methods section of the article. If the methods or included participants are different, the study will be included in this review. However, if the studies include similar populations, 1 study will be excluded after discussing among the authors. Three authors (NHM, NAFNA, and FNL) will retrieve the full texts and review them against the inclusion and exclusion criteria, along with the justifications. We will resolve any disagreement between the 2 reviewers via discussion or rechecking the full text. If a consensus is not reached, a third author (NAM) will deliberate.

**Figure 1:**
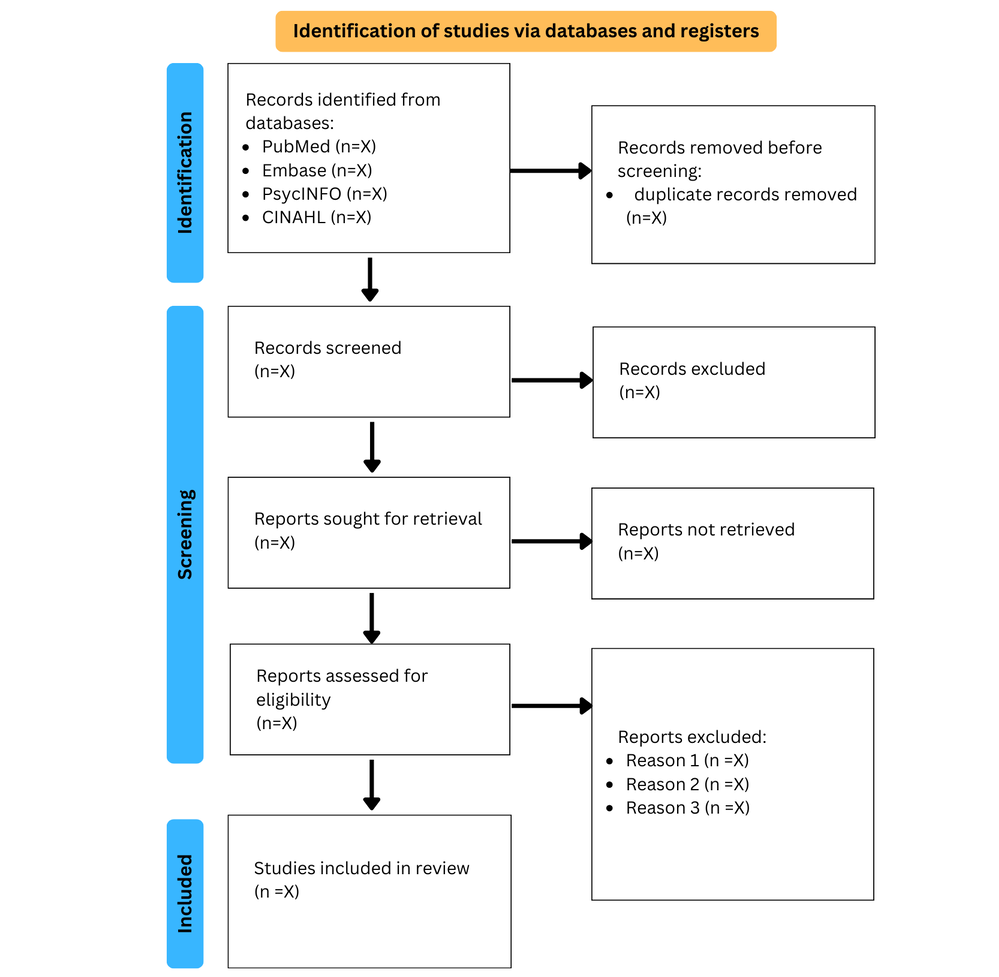
PRISMA (Preferred Reporting Items for Systematic Reviews and Meta-Analyses) flowchart.

We will record the selection process in sufficient detail to complete a PRISMA flowchart [15,16], as shown in Figure 1. This review will use the Mendeley reference management software (Elsevier) [17] to store, arrange, and manage all articles identified from the databases.

#### Data Extraction and Management

We will use a standardized data extraction form created in an online Microsoft Excel spreadsheet for study characteristics and outcome data. Two review authors (IAR and FNL) will independently extract the data from the included studies. If there is any disagreement on the data extraction, both authors will discuss and consult with another author (NAM). We will extract the following study characteristics from the included studies:

1. Article information: authors, year, title, country, and region.
2. Participants: total number of participants, age, sex, and history of psychiatric and any other morbidity disease.
3. Methods: study design and health care settings (primary care, hospital, or community).
4. Analysis: score from the questionnaire will be used to identify self-harm among respondents, prevalence will be reported, and factors associated with self-harm among HCWs will be discussed.

#### Quality Assessment

The study’s quality will be evaluated using the Joanna Briggs Institute’s (JBI’s) checklist by 3 review authors (NHM, TPNTBS, and NHJ) independently. If a consensus is not reached after discussion and reading back the article together, a third author (NAM) will deliberate. Multiple JBI scales will be used, as numerous types of observational studies will be included in this review, such as cross-sectional, case-control, and cohort studies. The overall appraisal of articles with the potential to be included will be defined either to include, exclude, or seek further information [18] based on the average answer evaluated by the 3 review authors. Each criterion with the answer “yes” will be accorded 1 point, and the cut-off point for being included, excluded, and seeking further information will be discussed among all authors. Articles with poor scores will be excluded from this review. The findings will be summarized in a table format. The details of this assessment, based on the criteria of the JBI checklist, will be listed in a Multimedia Appendix 1.

#### Statistical Analysis

Data will be analyzed using Stata (version 17.0; StataCorp) and RevMan (Cochrane) softwares. If there are missing data, the review author will contact the corresponding author to obtain the data. The study will be divided into 3 groups: overall, before, and after the COVID-19 pandemic, as we assume that there might be a difference in terms of overall prevalence as well as the risk factors associated with self-harm among HCWs.

We will segregate the data based on profession, such as physicians, nurses, and others. If the article does not mention the HCWs’ profession, the included sample will be referred to as “HCWs.” If the included articles mix the analysis for the professions, the term “HCW” will also be used. We will also perform a separate analysis based on the health settings (primary care, hospital, or community care). We will perform descriptive analysis, for example, sex, age, regions, and others. The risk factors associated with self-harm will be divided into a few domains: psychological factors (depression, anxiety, stress, and others), work-related factors (burnout, working hour or shift, satisfaction, engagement, workload, organizational conflict or culture, bullying, harassment, resources, service duration, profession, and others), and personal factors (age, relationship or marital status, income, support, health status, gender, self-conflict, life events, and others).

The overall pool prevalence of self-harm among HCWs will be calculated using a random effect model to allow heterogeneity across the included studies, and later divide the studies into regions. Freeman-Turkey transformation (arcsine square root transformation) will be applied to overcome the limitation of meta-analysis in prevalence studies, as some of the included articles will have very low or very high prevalence. This will possibly occur as we will collect articles from all types of study designs in this review. Having a wide range of prevalence values can result in disproportionately high weights due to the significant decrease in their inferred variance toward 0 [19]. Therefore, the Freeman-Turkey transformation will be used for pooling to obtain a synthesized point estimate of prevalence with a 95% CI [20].

A forest plot will be created for each pool estimate, and the distribution will be presented graphically. We will create a funnel plot if there are 10 or more studies available in 1 analysis to assess biases. We will assess funnel plot asymmetry visually and perform the Egger test to detect publication bias [21-23]. If the funnel plot is asymmetrical and the Egger test has a *P*<.05, it may suggest the presence of publication bias [23]. However, a symmetrical plot indicates the absence of publication bias in the included articles.

We will also conduct a meta-analysis by pooling the appropriate data using Cochrane’s statistical software, considering studies to be sufficiently similar in terms of population category, intervention, comparison, and outcome to observe the association between self-harm and the risk factors among HCWs. We will use a random effect model for pooled data. We will follow the strategies in the Cochrane Handbook for Systematic Reviews of Interventions for data management [21,22]. For multiple-arm studies, we will adjust the data following the methods described in the Cochrane Handbook for Systematic Reviews of Interventions [21,22]. Data analysis is expected to be completed by August 2025.

#### Assessment of Heterogeneity

We will visually inspect the forest plots for any evidence of heterogeneity. We will assess heterogeneity using both the Q test and *I*^2^ statistics. The *I*^2^ statistic will be used to quantify the impact of heterogeneity, while the Q test will be used to identify whether there is a significant heterogeneity of the included studies. We will set the significance level for the Q test at 0.01 [24] while the *I*^2^ level of 75% indicates a substantial degree of heterogeneity. We will adopt a higher threshold of *I*^2^ in this study due to the inherent heterogeneity in the included articles. If the I^2^ of the overall studies is less than 75%, the random effect model will be applied in the meta-analysis to overcome the inherent heterogeneity that might be highly present among different prevalent studies [25]. If significant heterogeneity is detected using the *I*^2^ index (>75%), we will investigate the potential sources of heterogeneity using subgroup analyses and meta-regression. The possible causes will be explored and evaluated for their methodological characteristics to determine whether the degree of heterogeneity can be explained by differences in those characteristics and if a meta-analysis is appropriate. The overall prevalence and 95% CI estimates of individual studies will be presented in forest plots. Forest plot with a *P*<.05 will be reported as statistically significant for the analysis [26].

### Dissemination

We will disseminate findings through flyers on the internet website. We will publish this review in an international open-access journal to have more citations and engagement regarding this issue. The findings will also be presented at conferences in both local and international settings to share our results. The preliminary findings will be presented to the clinicians, medical officers, and the Ministry of Health, Malaysia, through engagement, meetings, and forums. We will also produce plain language summaries that will be shared with the public through social media and health care websites. This review is expected to be completed and published by January 2026.

### Ethical Considerations

Because this review will only include published articles, ethics approval is not needed. All findings will be shared and disseminated at a local or international conference. The preliminary findings will be presented to psychiatrists or clinical practitioners of the Ministry of Health, Malaysia.

## Results

The article search will be performed in March 2025, and any articles published from inception until March 2025 will be included in this study before abstract and title screening. Data analysis is expected to be completed by June 2025, and the manuscript preparation is expected to be completed by July 2025. This review is expected to be completed and published by August 2025. Results on the global overall or pooled prevalence of self-harm among HCWs and the risk factors associated with it will be presented in this paper.

## Discussion

### Anticipated Findings

This review will highlight the updated overall prevalence of and factors associated with self-harm for better recognition of predisposing, precipitating, perpetuating, and protective factors for thoughts of suicide and self-harm among HCWs [26]. We believe that our review will be important for both clinicians and researchers, especially in controlling distress and potential harm among HCWs. The findings will note the differences between the conditions before and during the COVID-19 pandemic. Therefore, it will help clinical practices to advocate for safer workplaces, perhaps a different approach to treatment or intervention and strengthen the support for HCWs who are at risk of self-harm [27]. Furthermore, we will also observe how self-harm affects HCWs in different health care settings (primary care, hospital, and community), as the burden of work might be associated with self-harm among them. This issue will also influence the patient experience during their visit to different health care facilities [28]. The risk factors listed under the workplace-factor domain might also be associated with self-harm in different health care settings, for example, bullying [29] and long shift hours [26], among others. Therefore, we will perform a separate analysis for each health care setting, in addition to considering regions and types of professions.

This review will also be useful for institutions where HCWs work, such as hospitals, clinics, and local governments organizations, to improve public awareness of self-harm among HCWs. Such efforts are important for the benefit of patients and health services, as self-harm is a strong predictor of suicide [30], which has become a global health and social concern. Furthermore, programs such as counseling services and institutional interventions are needed to address this issue. Providing exercise facilities or aerobic exercise in the health care setting might help to decrease self-harm, as exercise has been shown to decrease depression [31]. A previous study reported the significant association between higher physical activity levels and reduced risk of depression (odds ratio [OR] 0.77, 95% CI 0.72-0.82) among HCWs. Interestingly, the association was also consistent in other subgroup analyses, such as age groups, gender, and regions. Furthermore, both low- and moderate-intensity physical activity showed the most significant protective effects against depression (low-intensity: OR 0.81, 95% CI 0.75-0.56; moderate-intensity: OR 0.79, 95% CI 0.72-0.87) [32]. In short, by understanding the protective mechanisms, spanning emotional, social, and environmental domains, it may help prevent or reduce the risk of self-harm and increase resilience among HCWs [33-35].

These important review findings will be shared publicly, and disseminated in publications and presented at conferences through poster or oral presentations. Furthermore, findings might be translated into practical recommendations for different health care settings to decrease the burden and self-harm cases among HCWs. The results of this study will delineate the differences by profession type, health care setting, and other relevant factors.

### Strengths and Limitations

A bias may have been introduced by limiting the language of the included articles to English due to resource constraints, such as a translational resource. To overcome this issue, we included 3 different databases during the article search to increase the number of articles retrieved. The most common study design included in this review was a cross-sectional study; therefore, it cannot demonstrate a causal relationship for self-harm among HCWs.

### Conclusions

Self-harm among HCWs is increasing annually, and multiple risk factors are associated with it. Therefore, this review will provide a comprehensive synthesis of the overall prevalence and describe the associated risk factors of self-harm among HCWs worldwide. It will also provide important information to inform practice in developing effective strategies for preventing and managing self-harm among HCWs. It will also provide useful information on the psychological support, create better clinical environments, and protect their mental health.

## Appendix

Multimedia Appendix 1 Proposed search terms for all the databases.

## Abbreviations

HCW: health care worker
JBI: Joanna Briggs Institute
OR: odds ratio
PRISMA: Preferred Reporting Items for Systematic Reviews and Meta-Analyses
