# Supplementary material for: Prevalence and Associated Risk Factors of Self-Harm Among Health Care Workers: Protocol for Systematic Review and Meta-Analysis
Source: JMIR Res Protoc. 2025 Sep 17;14:e67059. doi: 10.2196/67059 (PMC12489405; doi:10.2196/67059)
Supplement: Multimedia Appendix 1 [file resprot_v14i1e67059_app1.docx]

**S1 Table: Proposed Search Terms for all databases**

| **Databases** | **Search terms** |
| --- | --- |
| Pubmed/Embase/Cochrane/Psych Info/CINAHL | Healthcare worker*[Title/Abstract]) OR (Healthcare staff*[Title/Abstract] OR (Healthcare professional*[Title/Abstract] OR (Healthcare provider*[Title/Abstract] OR (Healthcare personnel*[Title/Abstract] OR (HCW*[Title/Abstract] OR (Nurse*[Title/Abstract] OR (Doctor*[Title/Abstract] OR (Physician [Title/Abstract] OR (Surgeon[Title/Abstract] OR (Therapist*[Title/Abstract] OR (Houseman[Title/Abstract] OR (House officer[Title/Abstract] OR (Medical officer[Title/Abstract] OR (nurs*[Title/Abstract] OR (midwi*[Title/Abstract] OR (registered nurse*[Title/Abstract] OR (RN[Title/Abstract] OR (Licensed practical nurse*[Title/Abstract] OR (LPN*[Title/Abstract] OR (Medical assistant*[Title/Abstract] OR (Paramedic*[Title/Abstract] OR (Pharmacist*[Title/Abstract] OR (Hospital Staff*[Title/Abstract] OR (General practitioner*[Title/Abstract] OR (GP[Title/Abstract] OR (Frontlin*[Title/Abstract] OR (Employer*[Title/Abstract] OR (Employee*[Title/Abstract] OR (Medical personnel*[Title/Abstract] OR (Medical professional*[Title/Abstract]  AND  Self overdos* [Title/Abstract] OR (Deliberate self-harm[Title/Abstract] OR (DSH[Title/Abstract] OR (self-inflict[Title/Abstract] OR (self-harm*[Title/Abstract] OR (self-mutilat*[Title/Abstract] OR (self-poison*[Title/Abstract] OR (self-injur*[Title/Abstract] OR (automutilat*[Title/Abstract] OR (ligature strangulate*[Title/Abstract] OR (self-hurt*[Title/Abstract] OR (self lacerat*[Title/Abstract] OR (self-hang*[Title/Abstract] OR (self-neglect*[Title/Abstract] OR (self-destruct[Title/Abstract] OR (self-immolat*[Title/Abstract] OR (self violen*[Title/Abstract] OR (self-injorious Behav*[Title/Abstract]  AND  Prevalen* [Title/Abstract] OR (incidence [Title/Abstract] OR (Number of cases [Title/Abstract] OR (risk factor* [Title/Abstract] OR (Associated factor*[Title/Abstract] OR (Predisposing factor*[Title/Abstract] OR (Factor*[Title/Abstract] OR (Predictor*[Title/Abstract] OR (Financial problem [Title/Abstract] OR (Marital status[Title/Abstract] OR (Family crisis[Title/Abstract] OR (Family problem [Title/Abstract] OR (Mental health problem[Title/Abstract] OR (Work related problem[Title/Abstract] OR (Physical health problem[Title/Abstract] OR (Substance use problem[Title/Abstract] OR (Alcohol problem[Title/Abstract] OR (Drug problem[Title/Abstract] OR (Poor support system[Title/Abstract] |
